# P62 Dental stewardship and antimicrobial resistance awareness in India: evidence from a systematic review

**DOI:** 10.1093/jacamr/dlaf230.069

**Published:** 2025-12-04

**Authors:** Rishal Dsouza, Rasha Abdelsalam Elshenawy

**Affiliations:** Department of Medicine, School of Health, Medicine and Life Sciences, University of Hertfordshire, UK, AL10 9AB; Department of Medicine, School of Health, Medicine and Life Sciences, University of Hertfordshire, UK, AL10 9AB

## Abstract

**Background:**

Antimicrobial resistance (AMR) is a critical global health threat, projected to cause up to 10 million deaths annually if left unaddressed.^1^ Antimicrobial stewardship (AMS) provides a structured framework to optimize prescribing practices and curb resistance.^2^ Dentistry plays a significant role in this crisis, with inappropriate antibiotic prescribing and widespread self-medication fuelling resistance.^3^ Dental stewardship programmes offer evidence-based frameworks to optimize antibiotic prescribing in dental practice and mitigate the AMR crisis through targeted interventions and improved prescribing protocols.^3^

**Objectives:**

To systematically review awareness, knowledge and perceptions of AMR among Indian dental professionals, and to examine AMS implementation, challenges, facilitators and recommendations for strengthening stewardship in dentistry.

**Methods:**

A systematic review was conducted in accordance with PRISMA guidelines. Literature was searched across PubMed, Scopus, CINAHL, Web of Science and Google Scholar for studies published between 2015 and 2025. Predefined search terms and Boolean operators were applied. Eligible studies included those involving dental professionals, students and dental settings in India, as well as AMR awareness, knowledge, perceptions of AMR, or AMS-related interventions. Non-human research and studies outside dentistry were excluded. Ethical approval was not required as no patient-identifiable data were used.

**Results:**

A total of 414 records were screened, with 14 studies meeting the inclusion criteria for quantitative and qualitative synthesis. Quantitative findings showed that all studies (100%) referenced guidelines and pathways, while 85.7% assessed prescribing practices and 57.1% included compliance monitoring. However, multidisciplinary teams and audit/feedback approaches were reported in only 7.1%, highlighting significant gaps in AMS implementation. Antibiotic prescribing patterns revealed Amoxicillin/Penicillin accounted for 62% of prescriptions, broad-spectrum agents 31% and others 7%. Ten studies reported problematic behaviours, with 80% empirical prescribing and 70% both prophylactic overuse and misuse of indications. Qualitative analysis identified four themes: poor AMR knowledge, a knowledge-practice disconnect, heterogeneity in prescribing practices and systemic educational/institutional barriers. Collectively, these findings highlight the urgent need for tailored stewardship strategies and integration of AMS into dental education and practice in India and promote dental stewardship in practice.

**Conclusions:**

This systematic review demonstrates a significant gap between theoretical AMR awareness and clinical practice among Indian dental professionals. Findings reveal critical deficiencies in prescribing knowledge, profound knowledge-practice disconnect and inappropriate antibiotic use, exacerbated by systemic barriers. Dental stewardship requires immediate multi-pronged strategies: educational reform, institutional antimicrobial stewardship programmes with monitoring and national evidence-based guidelines. Future research must focus on implementation science, developing practical real-world interventions, such as digital decision support tools, to bridge the identified knowledge-practice gap across dental stewardship settings effectively.
